# Identification of genes related to ketosis in dairy cows and establishment of early detection method for aryl hydrocarbon receptor gene

**DOI:** 10.3389/fvets.2026.1737345

**Published:** 2026-02-11

**Authors:** Yu Wang, Huizhong Zhang, Ying Li, Fengcai Guo, Jianyun Li, Wenlong Wang, Wenguang Zhang

**Affiliations:** 1 College of Veterinary Medicine, Inner Mongolia Agricultural University, Hohhot, China; 2 Inner Mongolia Engineering Research Center of Genomic Big Data for Agriculture, Hohhot, China; 3 Inner Mongolia Autonomous Region Center for Disease Control and Prevention, Hohhot, China; 4 College of Animal Science, Inner Mongolia Agricultural University, Hohhot, China

**Keywords:** biochemical index, disease-indicating gene, double standard curve, early warning, ketosis

## Abstract

**Introduction:**

Ketosis is a common metabolic disease in periparturient dairy cows. Monitoring and early warning based on blood indicators have become a key research focus in this field. Current diagnosis mainly relies on detecting plasma β-hydroxybutyric acid (BHBA). This study aims to explore the pathogenesis of ketosis at the blood genetic level, with the goal of achieving the early warning and prevention of the disease.

**Methods:**

According to China’s “gold standard” diagnostic method for dairy cow ketosis, blood samples were collected from different groups. Biochemical and oxidative stress indicators were then measured to evaluate organ function. Transcriptome sequencing analysis was performed on healthy cows and sick cows (both pre- and post-onset) to identify differentially expressed genes (DEGs). The functions of DEGs and their enriched pathways were analyzed through GO and KEGG pathway analyses. Weighted Gene Co-expression Network Analysis (WGCNA) was used to analyze the transcriptomic data, identify co-expression modules, and explore the relevance between these modules and the traits associated with ketosis occurrence. A dual standard curve method was established with the *AHR* gene as the core for early warning and detection. Then the specificity, sensitivity, and repeatability of the primers were validated. Finally, the method was applied to detect both positive samples (diseased cows) and healthy dairy cow samples to analyze the relative expression level of the *AHR* gene and the prediction accuracy.

**Results:**

Three biochemical indicators BHBA, non-esterified fatty acid (NEFA), and glucose (GLU) were identified as key diagnostic markers for ketosis in postpartum dairy cows. Changes in NEFA and GLU levels prior to parturition may indicate the risk of ketosis. A large number of DEGs were screened out, among which the *AHR* gene was identified as a candidate molecular marker gene for ketosis prediction. The early warning and detection method based on the *AHR* gene demonstrated high primer specificity, and the detection method itself exhibited excellent specificity, sensitivity, and repeatability. The prediction accuracy for healthy dairy cows reached 91.11%.

**Discussion:**

This study demonstrates that the detection method established based on the *AHR* gene is efficient and reliable, effectively distinguishing between early ketosis individuals and healthy ones based on gene expression differences. This study provides an efficient and reliable technical tool for the early warning of ketosis in dairy cows.

## 1 Introduction

In recent years, China’s dairy farming industry has experienced rapid development—with the national dairy cow stock increasing from 6.71 million head in 2015 to 8.52 million head in 2023, and the annual raw milk output rising from 35.31 million tons to 41.35 million tons over the same period—securing a pivotal position in animal husbandry. However, as dairy cattle populations increase, the industry is progressively transitioning toward intensification and large-scale operations. While this expansion creates growth opportunities, it also introduces significant challenges. Among these, ketosis represents the most severe metabolic disease, leading to neuroendocrine disorders and reproductive system complications. This condition substantially affects the reproductive performance of dairy cows, posing a serious threat to herd reproductive efficiency and economic returns.

Ketosis primarily affects high-producing dairy cows and is characterized by elevated levels of ketone bodies—acetone (Ac), acetoacetate (AcAc), and BHBA—in the bloodstream. The condition manifests in clinical and subclinical forms (1). Clinical signs include elevated ketone bodies in blood, milk, and urine, accompanied by a sweet acetone odor. Additional symptoms include depression, anorexia, weight loss, reduced milk production, and hypoglycemia. Surveys conducted in 2023 reported that ketosis incidence in high-yielding herds in China typically ranges from 2 to 20% of postpartum cows, with an increasing trend over the years. The highest prevalence was observed in cows with 3–7 parities (2). Poor feed quality, unbalanced diets, and insufficient roughage contribute to negative energy balance, thereby increasing ketosis prevalence in dairy cows.

Current methods for detecting ketosis include clinical diagnosis, laboratory testing, and genetic analysis. Clinical diagnosis is based on observations such as lethargy, appetite loss, weight reduction, decreased milk output, and dry feces. Laboratory methods include the ketone powder test, biochemical analysis, gas chromatography–mass spectrometry, blood ketone meter testing, and liver puncture (3). Researchers like Xu et al. (4) demonstrated the role of fibroblast growth factor-21 (FGF-21), a liver-synthesized protein, in energy metabolism and glucose homeostasis regulation. Serum FGF-21 levels in cows with ketosis, measured using ELISA kits, were found to correlate with energy metabolic status (4). Luke et al. (5)and Ho et al. (6) predicted serum concentrations of BHBA, NEFA, and urea nitrogen using MIR technology to assess metabolic disturbance risks during early lactation. Liu et al. (7) analyzed prenatal and postnatal NEFA components using GC–MS, proposing C18:1n9/C12:0 and C18:1n9/C22:1n9 ratios as potential ketosis indicators. Despite these advancements, gene-based detection methods for ketosis remain underdeveloped. This study aims to establish a gene-based early warning system for ketosis in dairy cows.

The aryl hydrocarbon receptor (*AHR*) is a versatile environmental sensor and transcription factor found throughout the body, responding to a broad spectrum of small molecules originating from the environment, our diets, host microbiota, and internal metabolic processes. Increasing evidence highlights *AHR*’s role as a critical regulator of numerous biological functions, such as cellular differentiation, metabolism, immune response and even Inflammation regulation (8). Shimba et al. (9, 10) found in the 3 T3-L1 cell model that overexpression of *AHR* could inhibit the differentiation of preadipocytes into mature adipocytes, while reduced *AHR* expression promotedadipogenesis and fat storage, suggesting that abnormal *AHR* expression may disrupt the homeostasis of adipose tissue and exacerbate energy metabolism imbalance. Wada et al. (11) demonstrated in a mouse model induced by high-fat diet (HFD) that liver-specific AHR knockout (AhR LKO) mice exhibited severe liver steatosis, inflammation, and damage. AHR can directly regulate the transcription of the cytokine suppressor of cytokine signaling 3 (Socs3) to inhibit its expression, thereby alleviating lipid toxicity and maintaining the balance of liver lipid metabolism. During ketosis in dairy cows, liver fat deposition and lipid toxicity are key pathological features. It is plausible that abnormal *AHR* function may exacerbate liver fat metabolism disorders and promote ketone body production through a similar mechanism (11). Cannon et al. (12) found in a mouse model of autoimmune hepatitis induced by concanavalin A that activation of *AHR* can alleviate liver inflammation injury by restricting the chemotaxis of pro-inflammatory immune cells, promoting the secretion of anti-inflammatory cytokines (such as IL-10), and inhibiting the production of pro-inflammatory factors (such as IL-6, IL-17A); at the same time, *AHR* can regulate the balance of Th17/Treg cells, increase the proportion of immunosuppressive Treg cells, and reduce the number of pro-inflammatory Th17 cells (12). Bowen et al. (13) pointed out in the bighorn sheep pneumonia model that in addition to its traditional detoxification function, *AHR* can also exert a protective effect by alleviating oxidative stress. In ketotic dairy cows, there is a significant level of oxidative stress, and AHR may participate in the regulation of the pathological process of ketosis by regulating the oxidative stress-related pathways (13). Dai et al. (14) found in Ashidan yaks that copy number variation (CNV) of the *AHR* gene is associated with growth traits, and changes in its expression level can affect fat deposition and energy metabolism. In summary, the existing literature has clearly demonstrated the crucial role of *AHR* in lipid metabolism, energy balance, immune inflammation, and oxidative stress regulation. These mechanisms are highly concordant with the core pathophysiological processes of ketosis in dairy cows, providing a robust theoretical foundation and theoretical support for in-depth exploration of the specific role and molecular mechanism of *AHR* in ketosis.

This study measured various biochemical parameters, including BHBA, GLU, NEFA, aspartate aminotransferase (AST), alanine aminotransferase (ALT), γ-glutamyl transferase (GGT), triglycerides (TG), total cholesterol (TC), total protein (TP), albumin (ALB), lactate dehydrogenase (LDH), and low-density lipoprotein cholesterol (LDL-C), as well as oxidative stress markers—reactive oxygen species (ROS), malondialdehyde (MDA), superoxide dismutase (SOD), glutathione peroxidase (GSH-Px), and catalase (CAT)—along with insulin (INS) levels in dairy cow blood to analyze their correlations with ketosis. Transcriptome analysis was performed to compare ketotic and healthy cows for the identification of differentially expressed genes (DEGs). Candidate genes potentially related to ketosis were subsequently selected as molecular markers to develop an early detection method.

## 2 Materials and methods

All protocols were approved by the Animal Care and Use Committee of Inner Mongolia Agricultural University, and all experiments adhered to animal safety policies. Dairy Holstein cows from a large intensive farm in Hohhot, Inner Mongolia, were selected for this study.

### 2.1 Biochemical indexes detection methods

The “gold standard” detection method involved serum collection, with BHBA levels measured using test strips and a blood ketone meter (093815701019, Abbott Diabetes Care, UK). GLU levels were determined by the enzymatic colorimetric method using a routine biochemical kit from Shenzhen Mindray Biomedical Electronics Co., Ltd., while NEFA levels were analyzed with the assay from the Beijing Huaying Biotechnology Research Institute. The serum concentrations of AST, ALT, GGT, TG, TC, TP, ALB, LDH, and LDL-C were measured by the enzymatic colorimetric method using biochemical kits from Shenzhen Mindray Biomedical Electronics Co., Ltd. Oxidative stress and insulin indexes, including SOD, CAT, MDA, GSH-Px, ROS and INS, were measured by the enzymatic colorimetric method using commercial Reagents assay kits (Beijing Huaying Biotechnology Institute, China).

### 2.2 Animals and samples

A total of 230 healthy Holstein cows from a large intensive farm in Hohhot, Inner Mongolia, were initially screened based on dry matter intake and clinical characteristics during the perinatal period. To ensure the accuracy of the study, cows with other diseases were excluded. From this initial pool, 30 ketotic cows and 30 healthy cows with similar parity and lactation days were selected for biochemical index screening. Blood samples were collected from the tail vein before morning feeding at two time points: 2 weeks before expected parturition and 2 weeks after parturition. Serum was separated by centrifugation at 4,000 rpm for 10 min at 4 °C, frozen in liquid nitrogen, and stored at −80 °C for biochemical analysis.

The “gold standard” diagnostic method was used to evaluate BHBA, NEFA, and GLU levels, determining and grouping experimental animals into four groups: prenatal healthy group (PHC), prenatal ketosis-prone group (PCK; cows that were healthy at the prenatal sampling but developed ketosis after delivery), postpartum healthy group (HC), and postpartum ketosis group (CK). Specifically, dairy cows with post-calving BHBA concentration exceeding 1.2 mmol/L were defined as the CK group.

For the subsequent transcriptome sequencing analysis, a subset of animals was carefully selected from the above groups. Six cows from the PCK group (healthy before parturition but developed ketosis after parturition) and four cows from the PHC group (healthy before parturition and remained healthy after) were chosen based on the most consistent and representative biochemical profiles. Blood samples were collected at two time points (2 weeks before and after parturition), resulting in 20 samples in total. To maintain RNA integrity, fresh blood was rapidly mixed with Trizol reagent and Buffer L9 from an RNA extraction kit at ratios of 1:3 and 1:2, respectively. The labeled samples were frozen in liquid nitrogen and stored at −80 °C for further use.

### 2.3 RNA library construction and sequencing

Based on the grouping results from the animal and sample section above, six cows in the PCK group (healthy before parturition but developed ketosis after parturition) and four cows in the PHC group (healthy before parturition and remained healthy after) were selected for whole-blood transcriptomics analysis. These 10 cows were sampled twice (2 weeks before and after delivery), providing 20 whole-blood samples for transcriptomic sequencing.

Total RNA was extracted and purified from each sample using “Whole Blood Total RNA Kit” (developed by Xinjing Biotechnology Co., Ltd.) following the manufacturer’s protocol. The RNA quantity and purity of each sample were quantified using NanoDrop ND-1000 (NanoDrop, Wilmington, Delaware, United States). RNA integrity was evaluated using Agilent 2100. Approximately 5 micrograms of total RNA was used to remove ribosomal RNA according to the instructions of the Ribo-ZeroTM rRNA Removal Kit (Illumina, San Diego, California, United States). The remaining RNA fragments were reverse transcribed using the RNA-seq library preparation kit (Illumina) to form the final cDNA. Finally, paired-end sequencing was performed on the Illumina Hiseq 4000 according to the protocol recommended by the supplier.

### 2.4 RNA extraction

Using the “Whole Blood Total RNA Kit” (Xinjing Biotechnology Co., Ltd.), we extracted RNA from blood samples of ketotic and healthy dairy cows at pregnancy and postpartum stages.

### 2.5 Real-time fluorescent quantitative PCR

For the first reaction, a 20 μL reaction system was prepared following the instructions of the ChamQ Universal SYBR qPCR Master Mix. The mixture included 10 μL of 2 × ChamQ Universal SYBR qPCR Master Mix, 0.4 μL each of upstream and downstream primers, 2 μL of the DNA template, and 7.2 μL of ddH₂O to achieve the total volume. The reaction followed a three-step protocol: (1) pre-denaturation at 95 °C for 30 s; (2) 40 cycles of denaturation at 95 °C for 10 s, followed by annealing at 60 °C for 30 s; and (3) a melting curve analysis program with conditions of 95 °C for 15 s, 60 °C for 60 s, and 95 °C for 15 s. Once the primers were confirmed to match well and produce an amplification curve, the reaction system was optimized.

During the optimization process, a positive plasmid standard with a concentration of 1 × 10^9^ copies/μL was used as the template, and qPCR conditions were tested using the SYBR Green dye method. Five primer concentration gradients were set: 10 μmol/L, 5 μmol/L, 2.5 μmol/L, 1.25 μmol/L, and 0.625 μmol/L, along with four annealing temperatures: 54 °C, 56 °C, 58 °C, and 60 °C. Each sample was tested in triplicate, and a template blank control group was established for each combination of temperature and concentration. The specificity of the coding sequences (CDs) of nine genes, including *AHR* and *KMT2A*, as well as the internal reference gene *GAPDH*, was verified using the NCBI BLAST tool to ensure high sequence matching and no significant cross-matching with other genes. Primers were designed using Primer 5 software, targeting amplified fragment lengths of 80–200 bp, with primer lengths of 18–28 bp and GC content controlled between 40 and 60%. The primers were synthesized by a biotechnology company (Table 1).

**Table 1:** Primer sequences.

Primers	Primer pairs	Primer sequences (5′-3′)	GenBank no	Product size (bp)
AHR	Sense primer	GGCATAGAGACCGACTTAATACAGAAC	NM_001206026.1	119
	Antisense primer	GAGATAACTGACACTGAGCCTAAGAAC		
KMT2A	Sense primer	CCAGCCTCTCAGTCGTTGTT	XM_059875015.1	137
	Antisense primer	ATGAGTAGGCCCGAAGGACT		
RASGRP3	Sense primer	GATGAGATGATGGCTTACTTCCTGAG	XM_024998733.2	112
	Antisense primer	CACAGAAGGTTGGCTTGAGATAGG		
BoLA	Sense primer	GAACCTCCTCAACCCTCCATCC	XM_059880385.1	121
	Antisense primer	CTGTTTGACCTGAGTGCTTCTTCC		
CPXM2	Sense primer	AACCATGACATCCGAACAGCC	NM_001206057.2	169
	Antisense primer	GGTTGGTTTTGCTGAGCGTGAA		
ITPR2	Sense primer	GGACAACTGCTCACCTACAATACC	NM_174369.2	113
	Antisense primer	GGTTCAGGACGGTCACAATGC		
DUSP4	Sense primer	TCTATTCCGCCGTCATCGTCTAC	NM_001206953.2	149
	Antisense primer	GAACCTCTCATAGCCACCTTTCAG		
CXCR3	Sense primer	TGAGACCTACTTCTGCTGTACTTCC	NM_001011673.1	134
	Antisense primer	CGACTGCCACGATGCCATTAC		
ITPR1	Sense primer	TGAATCCAACCAATGCCGACATC	NM_001435139.1	126
	Antisense primer	AGACCCATACTTCTTCCTCATCCTC		
GAPDH	Sense primer	CAAGTTCAACGGCACAGTCAAGG	NM_001034034.2	130
	Antisense primer	CTCCACCACATACTCAGCACCAG		

### 2.6 Construction of prokaryotic expression vectors

The recombinant plasmids pMD-AHR and pMD-GAPDH were constructed using the following steps. Reagents were added to PCR tubes, and amplification was performed in a programmed thermal cycler. The PCR products were analyzed by 2% agarose gel electrophoresis, and the target gene fragments were recovered. The recovered fragments were mixed with the pMD-19 T vector, and ligation was performed overnight at room temperature with ligation buffer. The ligation products were transformed into Trans1 T1 competent cells, followed by ice incubation, heat shock, cooling, and subsequent culturing in LB medium without antibiotics. The bacterial suspension was spread on LB agar plates containing ampicillin, and colonies resistant to ampicillin were selected after incubation. Colonies were picked and inoculated into liquid LB medium containing ampicillin, followed by PCR amplification and electrophoresis for verification. Plasmids were extracted, sequenced by BGI, and the plasmid concentration was measured. Based on the sequencing results, the plasmid concentration was adjusted to 1 × 10^9^ copies/μL for subsequent experiments.

### 2.7 Data statistics

Data were organized and analyzed using GraphPad Prism 8 software, with all results expressed as “Mean ± SD.” Statistical differences were analyzed using one-way ANOVA. Raw data from the Illumina HiSeq platform were filtered to remove low-quality reads and adapters. Differentially expressed genes (DEGs) between groups were identified using the DESeq2 package. Primers were designed using *GAPDH* as the reference gene, and relative gene expression was calculated using the 2^−ΔΔCt^ method after qRT-PCR. GO and KEGG enrichment analyses of DEGs were conducted using the DAVID online tool, while the WGCNA package was employed to construct gene co-expression networks. Positive plasmid standards of pMD-AHR and pMD-GAPDH were used as templates, with nuclease-free water serving as the negative control.

## 3 Results

### 3.1 Changes in blood biochemical indicators and screening of diagnostic markers for dairy cow ketosis

**Figure 1:**
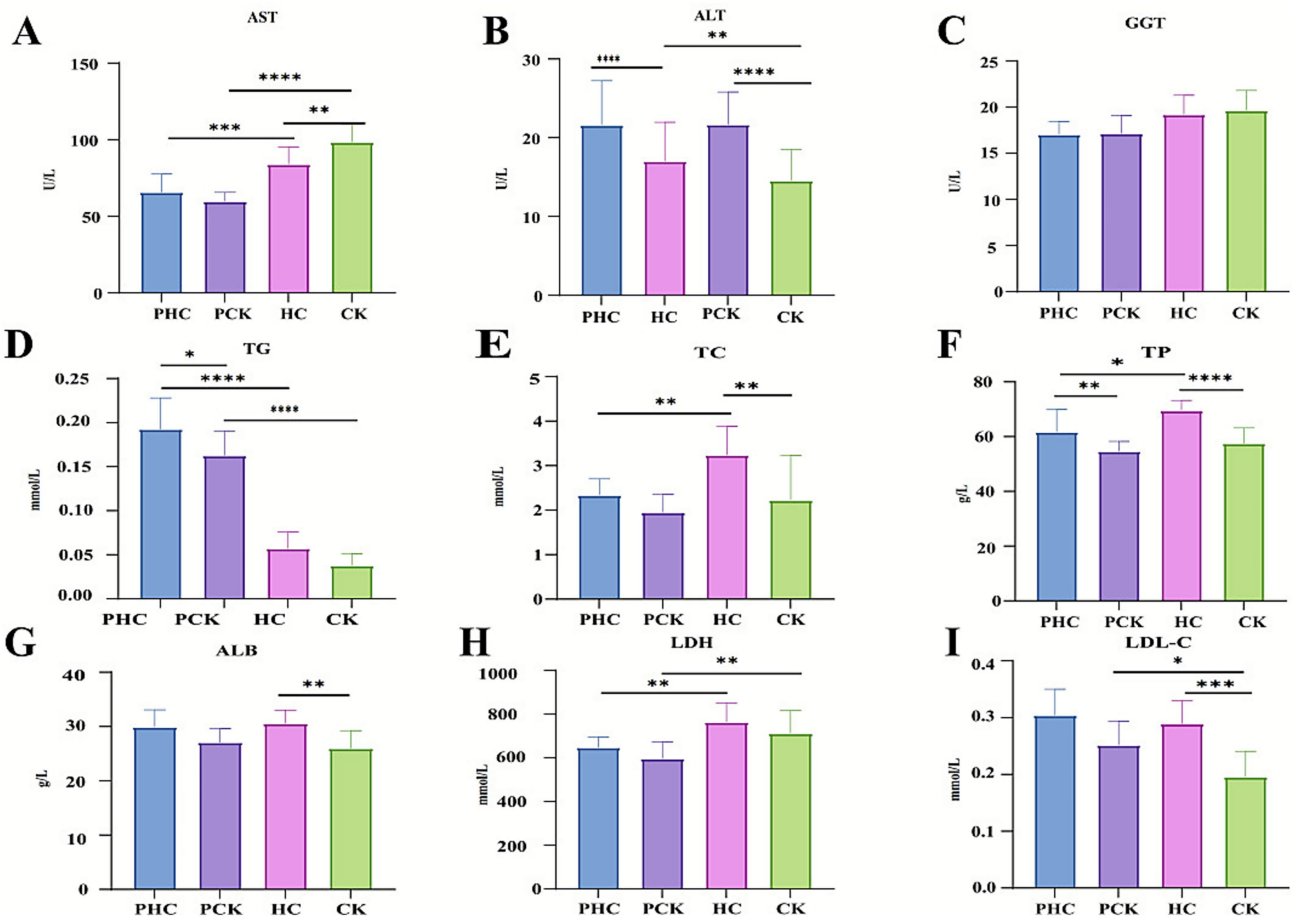
Biochemical indicator test. **(A)** Aspartate aminotransferase. **(B)** Alanine aminotransferase. **(C)** Gamma-glutamyl transferase. **(D)** Triglycerides. **(E)** Total cholesterol. **(F)** Total protein. **(G)** Albumin. **(H)** Lactate dehydrogenase. **(I)** Low-density lipoprotein cholesterol. *P < 0.05, **P < 0.01, ***P < 0.001, ****P < 0.0001. All data are presented as “Mean ± SD.”

Based on the biochemical test results, group comparisons revealed the following:

- PCK group vs. PHC group: The PCK group showed a significant decrease in TG content (*p* < 0.05, Figure 1D) and a significant reduction in ALT and TP levels (*p <* 0.01, Figures 1B,E).
- CK group vs. HC group: The CK group exhibited a significant increase in AST content (*p <* 0.01, Figure 1A), while TC, ALT, ALB, LDL-C, and TP levels significantly decreased (*p <* 0.01, Figures 1B,E–G,I).
- HC group vs. PHC group: The HC group demonstrated a significant increase in TP content (*p* < 0.05, Figure 1F) and significant increases in AST, TC, and LDH levels (*p <* 0.01, Figures 1A,E,H). Conversely, TG levels significantly decreased (*p <* 0.01, Figure 1D).
- CK group vs. PCK group: The CK group displayed significantly increased AST and LDH levels (*p* < 0.01, Figures 1A,H), a significant decrease in LDL-C content (*p <* 0.05, Figure 1I), and significant reductions in ALT and TG levels (*p <* 0.01, Figures 1B,D). There were no significant differences in GGT levels in the blood among the four groups of dairy cows (*p* > 0.05, Figure 1C).

Prior to parturition, dairy cows already exhibited metabolic abnormalities related to the risk of ketosis (such as decreased levels of TG, ALT, and TP). Postpartum, ketosis further led to liver function damage (with elevated AST) and metabolic disorders (significant reductions in TC, ALB, LDL-C, TP). Even for healthy cows, there were observed physiological and metabolic fluctuations during the perinatal period. Overall, indicators such as TG, ALT, and TP can serve as early warning signals for ketosis, while AST and others reflect liver damage during the progression of the disease.

### 3.2 Screening of markers of oxidative stress and insulin for dairy cow ketosis

**Figure 2:**
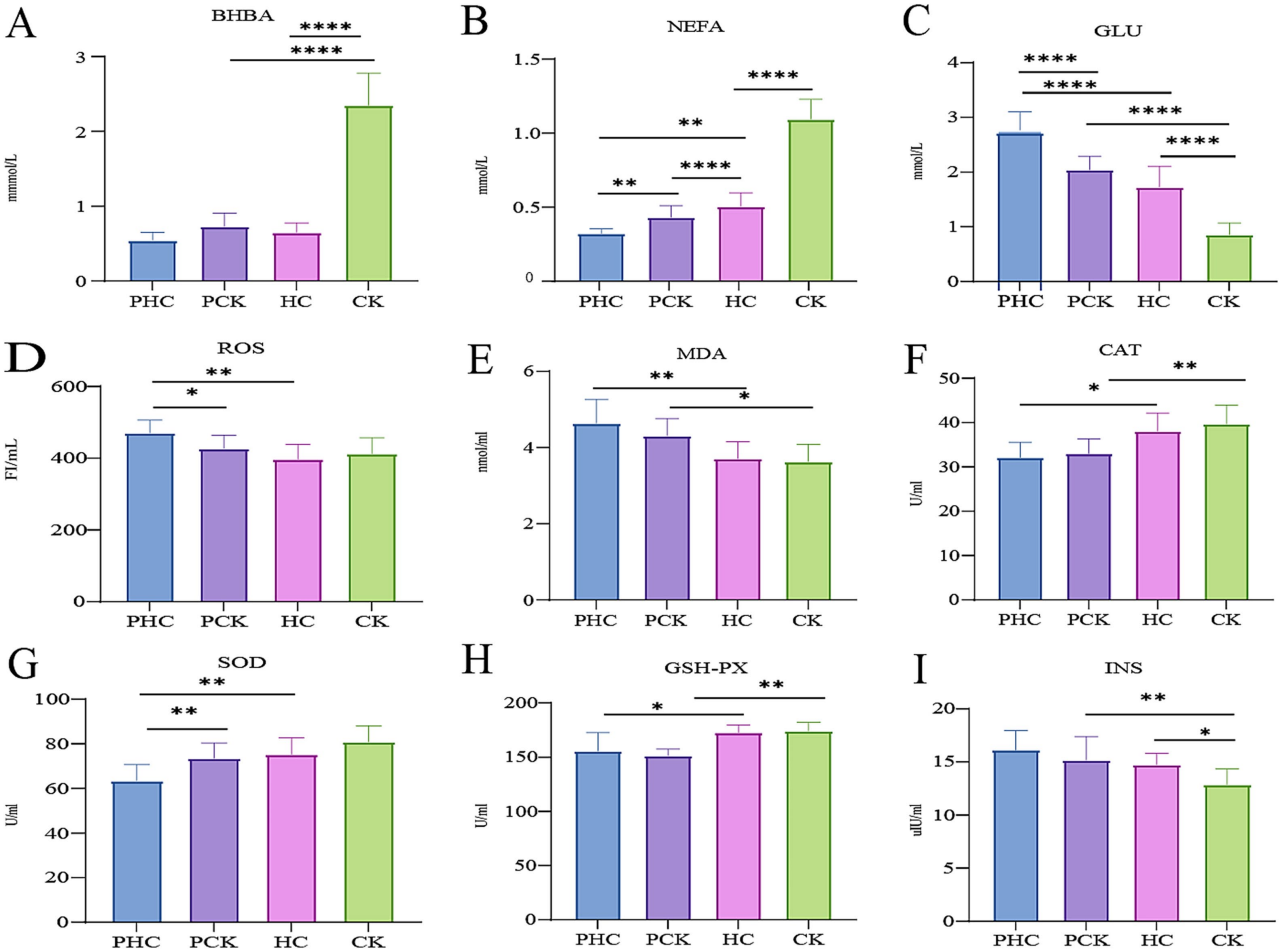
“Gold standard,” oxidative stress, and insulin test results. **(A)** Beta-Hydroxybutyrate (BHBA) levels. **(B)** Non-Esterified Fatty Acids (NEFA) levels. **(C)** Glucose (GLU) levels. **(D)** Reactive Oxygen Species (ROS) levels. **(E)** Malondialdehyde (MDA) levels. **(F)** Catalase (CAT) activity. **(G)** Superoxide Dismutase (SOD) activity. **(H)** Glutathione Peroxidase (GSH-Px) activity. **(I)** Insulin (INS) levels. *p < 0.05, **p < 0.01, ***p < 0.001, ****p < 0.0001. All data are presented as “Mean ± SD.”

Ketosis diagnosis was based on the biochemical “gold standard” markers. The BHBA levels in the PHC, PCK, and HC groups were within the healthy range, while BHBA levels in the CK group significantly increased (Figure 2A), consistent with ketosis characteristics. NEFA levels were higher in the CK group compared to the PHC, PCK, and HC groups (Figure 2B), aligning with increased NEFA in ketosis. NEFA levels in the PCK group were significantly higher than in the PHC group (*p <* 0.01). Postpartum GLU levels showed a significant decline compared to prepartum levels, with significant differences between the CK and HC groups (*p <* 0.01). GLU levels in the CK group significantly decreased (Figure 2C), indicating abnormal glucose metabolism consistent with ketosis diagnosis. NEFA and GLU level changes before parturition could serve as potential diagnostic markers for predicting ketosis.

Five oxidative stress markers were measured:

- Compared to the PHC group, ROS levels significantly decreased (*p* < 0.05), and SOD levels significantly increased (*p <* 0.01) in the PCK group (Figures 2D,G).
- In the HC group, CAT, GSH-Px, and SOD levels significantly increased (Figures 2F–H), while MDA and ROS levels significantly decreased (*p <* 0.01, Figures 2D,E).
- In the CK group, CAT and GSH-Px levels were significantly higher (*p <* 0.01), and MDA levels were significantly lower (*p <* 0.05) compared to the PCK group (Figures 2E,F,H).
- INS levels significantly decreased in the CK group compared to the PCK group (*p <* 0.01, Figure 2I) and were significantly lower than in the HC group (*p <* 0.05). ROS and SOD levels could serve as oxidative stress markers for early ketosis warning.

As the “gold standard” for diagnosing ketosis in dairy cows, BHBA showed a significant increase only in the CK group, consistent with the clinical characteristics. NEFA increased in both the PCK and CK groups, while GLU significantly decreased in the postpartum period, especially in the CK group. This indicates that the prepartum changes in NEFA and GLU can serve as early predictive indicators of ketosis. In the context of oxidative stress, the PCK group showed compensatory antioxidant responses such as decreased ROS and increased SOD, while the antioxidant capacity of healthy postpartum cows (HC) further enhanced and oxidative damage was reduced. In contrast, although antioxidant enzymes (CAT, GSH-Px) increased in the CK group, INS significantly decreased, suggesting a metabolic-oxidative imbalance. In summary, the changes in indicators such as NEFA, GLU, ROS, and SOD before parturition have predictive value for ketosis.

### 3.3 Transcriptomic analysis of whole blood in dairy cow ketosis

**Figure 3:**
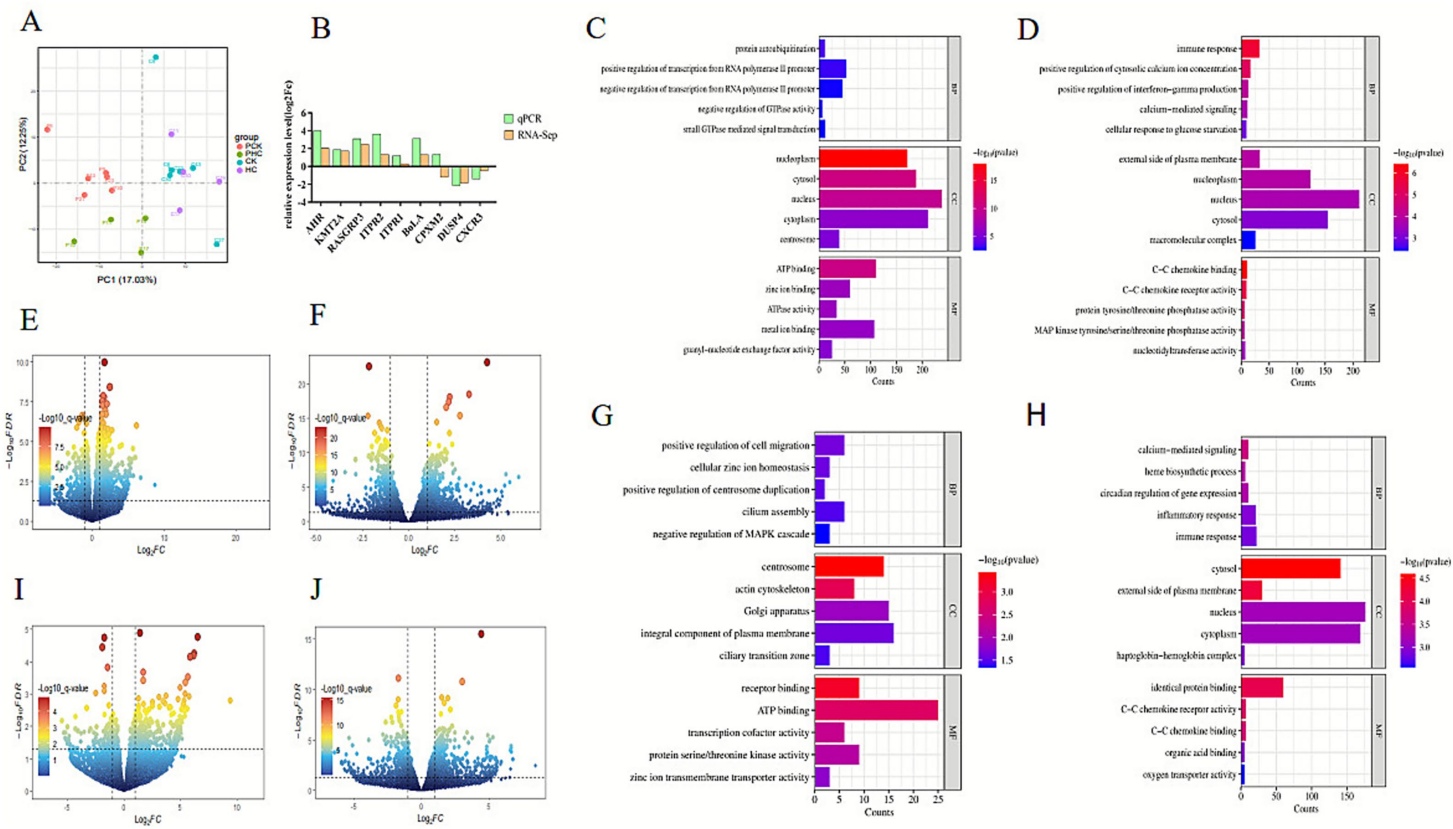
Transcriptomics analysis results. **(A)** PCA analysis of transcriptomic data illustrating sample clustering. **(B)** Validation of DEGs using qPCR. **(C,D,G,H)** GO enrichment analysis results depicting significant biological processes, cellular components, and molecular functions associated with DEGs. **(E,F,I,J)** Volcano plots of DEGs highlighting significantly upregulated and downregulated genes. In this figure, the corresponding log_2_FoldChange values for the three genes *AHR*, *RASGRP3*, and *KMT2A* are 2.05, 2.49, and 1.74 respectively; the *p*-value values are 9.17 × 10^−5^, 3.93 × 10^−9^, and 1.16 × 10^−10^, respectively.

To explore mRNA expression patterns among the PCK, PHC, CK, and HC groups, reads were mapped to the reference genome, and principal component analysis (PCA) was performed using the plotPCA tool in the DESeq2 software. The results indicated that mRNA expression patterns in the experimental groups remained consistent before and after the peripartum period, whereas the healthy control group exhibited greater inter-individual variability in mRNA expression patterns after parturition, as shown in Figure 3A.

A comparison of gene expression variations among groups revealed 1,153 differentially expressed genes (DEGs) in the PHC and PCK groups, with 246 downregulated and 907 upregulated genes. Notably, AHR, RASGRP3, and KMT2A showed the most pronounced differences in expression. Between the PCK and CK groups, 1,165 DEGs were identified, with 714 upregulated and 451 downregulated genes in the CK group. Between the CK and HC groups, 345 DEGs were identified, including 249 upregulated and 96 downregulated genes in the CK group. Between the PHC and HC groups, a total of 977 DEGs were identified, with 509 upregulated and 468 downregulated genes in the HC group, as shown in Figures 3E,F,I,J.

To verify the reliability of the transcriptomic sequencing data and bioinformatics analyses, qPCR validation was performed on 9 selected DEGs. As shown in Figure 3B, the trends in gene expression observed in RNA-seq results were consistent with those obtained from qPCR, indicating high reliability and accuracy of the RNA-seq data in this study. Differential analysis between the PHC and PCK groups showed significant expression differences for *AHR*, *RASGRP3*, and *KMT2A*, with log2FoldChange values of 2.05, 2.49, and 1.74, and *p*-values of 9.17 × 10^−5^, 3.93 × 10^−9^, and 1.16 × 10^−10^, respectively. These genes may serve as candidate markers for predicting ketosis risk.

Transcriptomic data across the prepartum to postpartum stages were analyzed, and DEGs were annotated using the DAVID website. Enrichment of DEGs in various GO terms was compared across groups. KEGG analysis was performed using *p*-value and count numbers as significance criteria. Between the PCK and PHC groups, DEGs were significantly enriched in 44 KEGG pathways involving metabolism and energy balance, including the Cushing syndrome pathway. Between the PCK and CK groups, DEGs were enriched in 37 KEGG pathways, including transcriptional misregulation in cancer. Between the CK and HC groups, DEGs were enriched in 5 KEGG pathways related to metabolism, endocrine function, immune response, and disease pathways. Between the PHC and HC groups, 23 KEGG pathways were enriched, primarily involving signal transduction, cell communication, metabolic pathways, and substance synthesis, as shown in Figures 3C,D,G,H.

**Figure 4:**
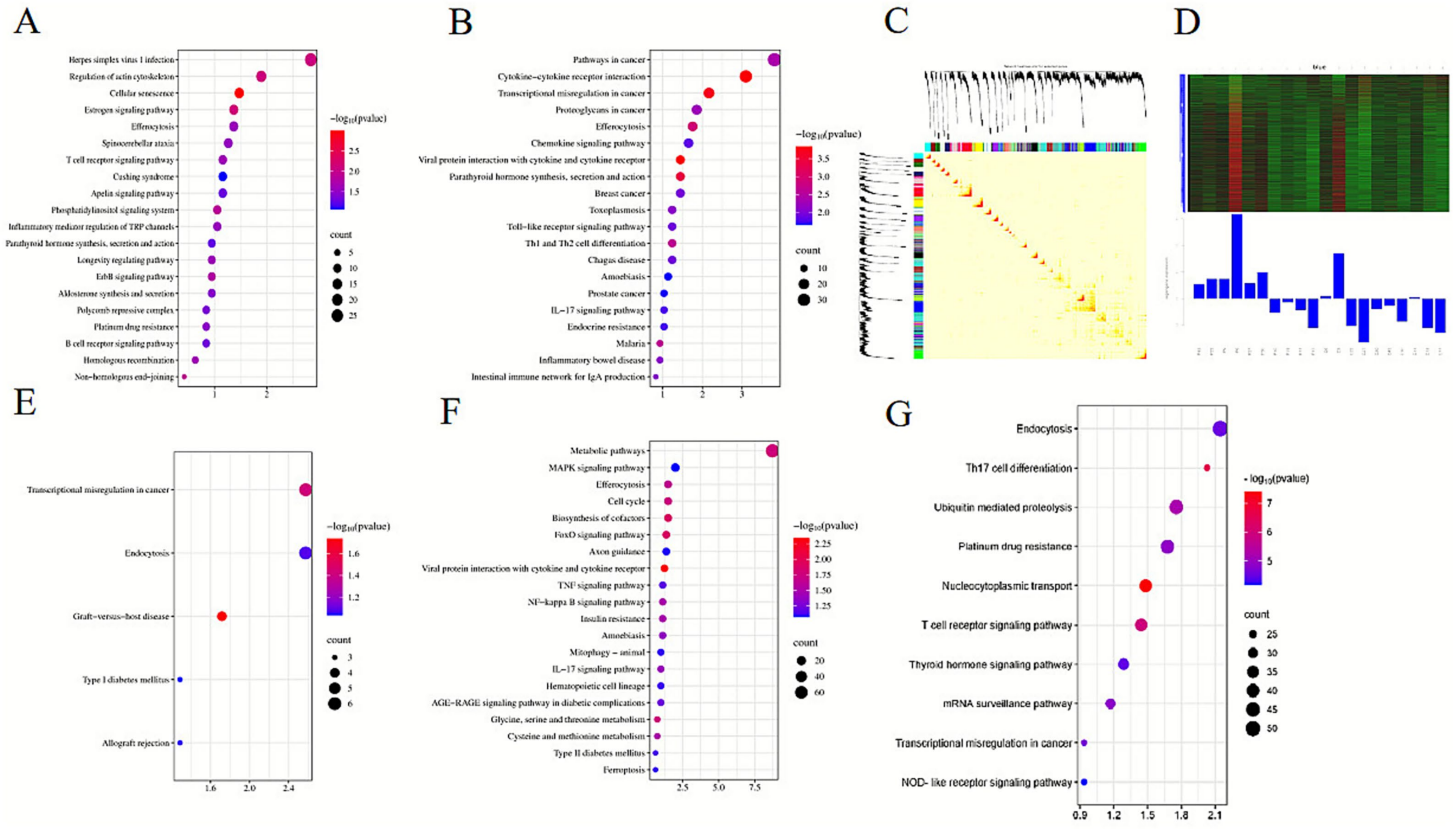
Enrichment analysis results. **(A,B,E,F)** KEGG pathway enrichment analysis for DEGs, highlighting pathways associated with metabolism, immune response, and signaling. **(C,D)** WGCNA analysis results, including module-trait relationships and module clustering. **(G)** KEGG pathway enrichment results for genes in the Blue module, illustrating pathways linked to Th17 cell differentiation and other critical processes.

Transcriptomic data were further analyzed using WGCNA, with the Blue module showing the highest correlation with traits predictive of ketosis (correlation coefficient *r* = 0.65, *p*-value = 0.002), as shown in Figures 4C,D,G. KEGG analysis revealed significant enrichment of Blue module genes in pathways related to intracellular transport and processing, as shown in Figures 4A,B,E,F. Notably, genes in the Blue module were significantly associated with the Th17 cell differentiation pathway. Among 24 genes regulating Th17 cell differentiation, the expression of the *AHR* gene showed a high correlation with the module characteristic value. Changes in *AHR* gene expression were closely associated with the occurrence of ketosis and may serve as a molecular marker for predicting ketosis in dairy cows.

### 3.4 Establishment of the early warning detection method for the *AHR* gene in dairy cow ketosis

**Figure 5:**
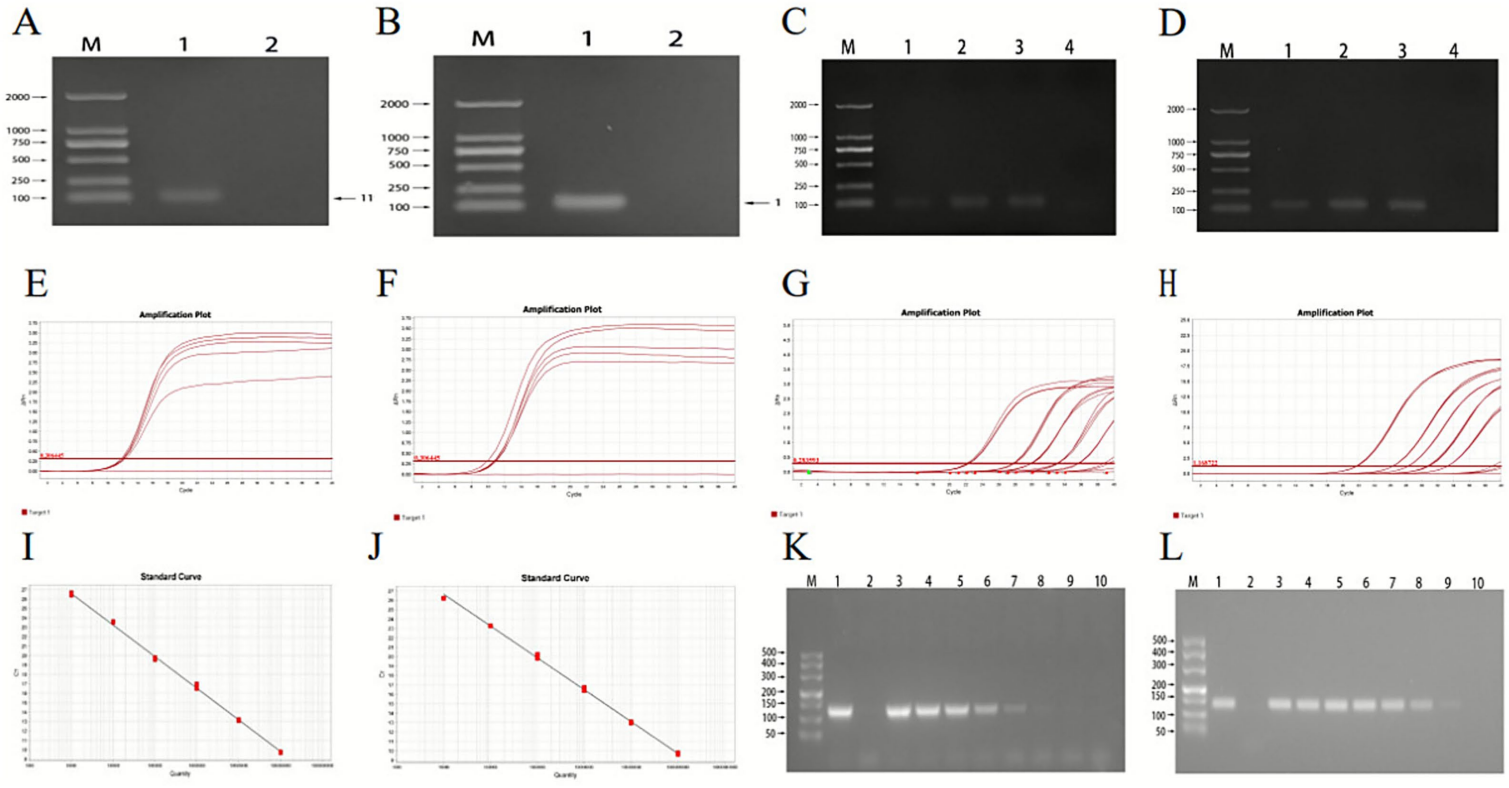
Primer specificity and sensitivity results of PCR. **(A–D)** Primer specificity maps. **(E)** Optimization of qPCR reaction conditions for pMD-AHR primers. **(F)** Optimization of qPCR reaction conditions for pMD-GAPDH primers. **(G)** Results of pMD-AHR qPCR sensitivity experiments. **(H)** Results of pMD-GAPDH qPCR sensitivity experiments. **(I)** pMD-AHR standard curves. **(J)** pMD-GAPDH standard curves. **(K,L)** Sensitivity experiment results for PCR. The template in **(K)** is the pMD-AHR plasmid; the template in **(L)** is the pMD-GAPDH plasmid. M represents the DL500 DNA marker. Lane 1 is the positive control (dairy cow ketosis DNA); lane 2 is the negative control (sterile water). Templates in lanes 3–10 correspond to recombinant plasmids at concentrations of 1 × 10^8^, 1 × 10^7^, 1 × 10^6^, 1 × 10^5^, 1 × 10^4^, 1 × 10^3^, 1 × 10^2^, and 1 × 10^1^ copies/μL, respectively. The standard curve equation for pMD-AHR: *y* = −3.352*x* + 39.989, with *R*^2^ of 0.998 and amplification efficiency of 98.8%. The standard curve equation for pMD-GAPDH: *y* = −3.384*x* + 40.18, with R^2^ of 0.996 and amplification efficiency of 97.5%.

The specificity of the primers designed for this experiment was first verified. Using the genomic cDNA of dairy cows with ketosis as a template, specific amplification of the *AHR* and *GAPDH* genes was conducted via PCR. Gel electrophoresis confirmed that the amplified sequences for both genes ranged between 100 and 250 bp, matching the expected fragment sizes. No non-specific amplification products were observed, indicating high primer specificity (Figures 5A,B).

To optimize qPCR reaction conditions, the primer concentrations were evaluated. For the pMD-AHR gene, optimal amplification was achieved at a primer concentration of 5 μmol/L, as shown in Figure 5E. For the pMD-GAPDH gene, the optimal primer concentration was 2.5 μmol/L (Figure 5F). PCR amplification of the bacterial solutions containing the pMD-AHR and pMD-GAPDH recombinant plasmids, using specific primers, confirmed that the target band sizes were consistent with expected values (100–250 bp), as shown in Figures 5C,D.

The prepared recombinant plasmids were adjusted to a working concentration based on calculated copy numbers, then subjected to a 10-fold gradient dilution from 1 × 10^9^ to 1 × 10^4^ copies/μL. These dilutions served as templates to construct qPCR standard curves under optimized conditions. For pMD-AHR, the standard curve equation was:

*y* = −3.352*x* + 39.989, with an *R*^2^ of 0.998 and amplification efficiency of 98.8% (Figure 5I).

For pMD-GAPDH, the standard curve equation was:

*y* = −3.384*x* + 40.18, with an *R*^2^ of 0.996 and amplification efficiency of 97.5% (Figure 5J).

The amplification curve of pMD-AHR indicated successful amplification at concentrations ranging from 10^6^ to 10^2^ copies/μL, with two valid results observed in three parallel wells at a concentration of 10 copies/μL. Similarly, for pMD-GAPDH, valid results were obtained at concentrations between 10^6^ and 10^2^ copies/μL, with two valid results at 10 copies/μL (Figures 5G,H).

Ordinary PCR sensitivity detection using the same recombinant plasmids revealed that the lowest detection limit for *AHR* primers was 1 × 10^4^ copies, while the qPCR method improved sensitivity approximately 1,000-fold. For *GAPDH* primers, the lowest detection limit was 1 × 10^3^ copies, with the qPCR method demonstrating approximately 100-fold greater sensitivity (Figures 5K,L).

### 3.5 Diagnostic experiments of clinical blood sample early warning detection

The diagnostic experiments conducted on clinical blood samples validated the specificity and repeatability of the qPCR early warning detection method for the *AHR* gene.

**Figure 6:**
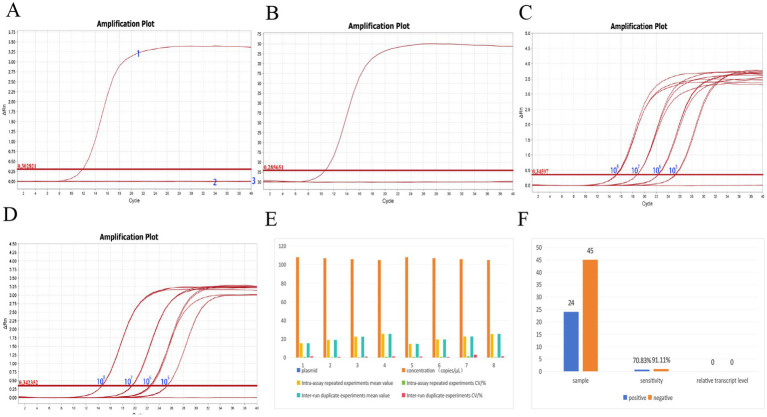
Results of diagnostic assay. **(A)** Results of pMD-AHR qPCR specificity assay. **(B)** Results of pMD-GAPDH qPCR specificity assay. **(C)** Results of pMD-AHR qPCR reproducibility experiments. **(D)** Results of pMD-GAPDH qPCR reproducibility experiments. **(E)** qPCR repeatability experiment data. **(F)** Experimental results of the *AHR* early warning detection method. 1 and 2 indicate positive plasmids; 3 represents the enzyme-free water negative control sample.

Firstly, using the positive standard plasmids of pMD-AHR and pMD-GAPDH as templates, qPCR specificity tests were performed. For the pMD-AHR plasmid, results showed that only the pMD-AHR plasmid yielded a positive amplification, while all other templates were negative, confirming its specificity (Figure 6A). Similarly, the pMD-GAPDH plasmid exhibited positive amplification, with all other plasmids being negative, demonstrating high specificity for this method (Figure 6B).

Secondly, recombinant plasmids with four gradient concentrations—1 × 10^8^ copies/μL, 1 × 10^7^ copies/μL, 1 × 10^6^ copies/μL, and 1 × 10^5^ copies/μL—were used as templates for amplification with both primer sets. Repeatability tests were conducted using different batches of qPCR enzymes. The results showed that the coefficient of variation (CV%) among groups for pMD-AHR qPCR was less than 1.344%, and the CV% within groups was less than 0.475%, indicating high repeatability. The amplification curve for pMD-AHR qPCR is presented in Figure 6C. For pMD-GAPDH qPCR, the CV% among groups was less than 1.268%, and the CV% within groups was less than 3.166%, confirming good repeatability and stability (Figure 6D). Repeatability test data are summarized in Figure 6E.

Testing of 24 positive samples revealed a concordance rate of 70.83%, with relative *AHR* gene expression ranging from 1.9571 to 15.5321. For 45 negative control samples, the concordance rate was 91.11%, with relative *AHR* expression values ranging between 0.4341 and 1.7924 (Figure 6F). These results demonstrate the potential of the *AHR* early warning detection method for clinical applications.

### 3.6 Correlation analysis of *AHR* gene with blood key biochemical indicators, antioxidant indicators

**Figure 7:**
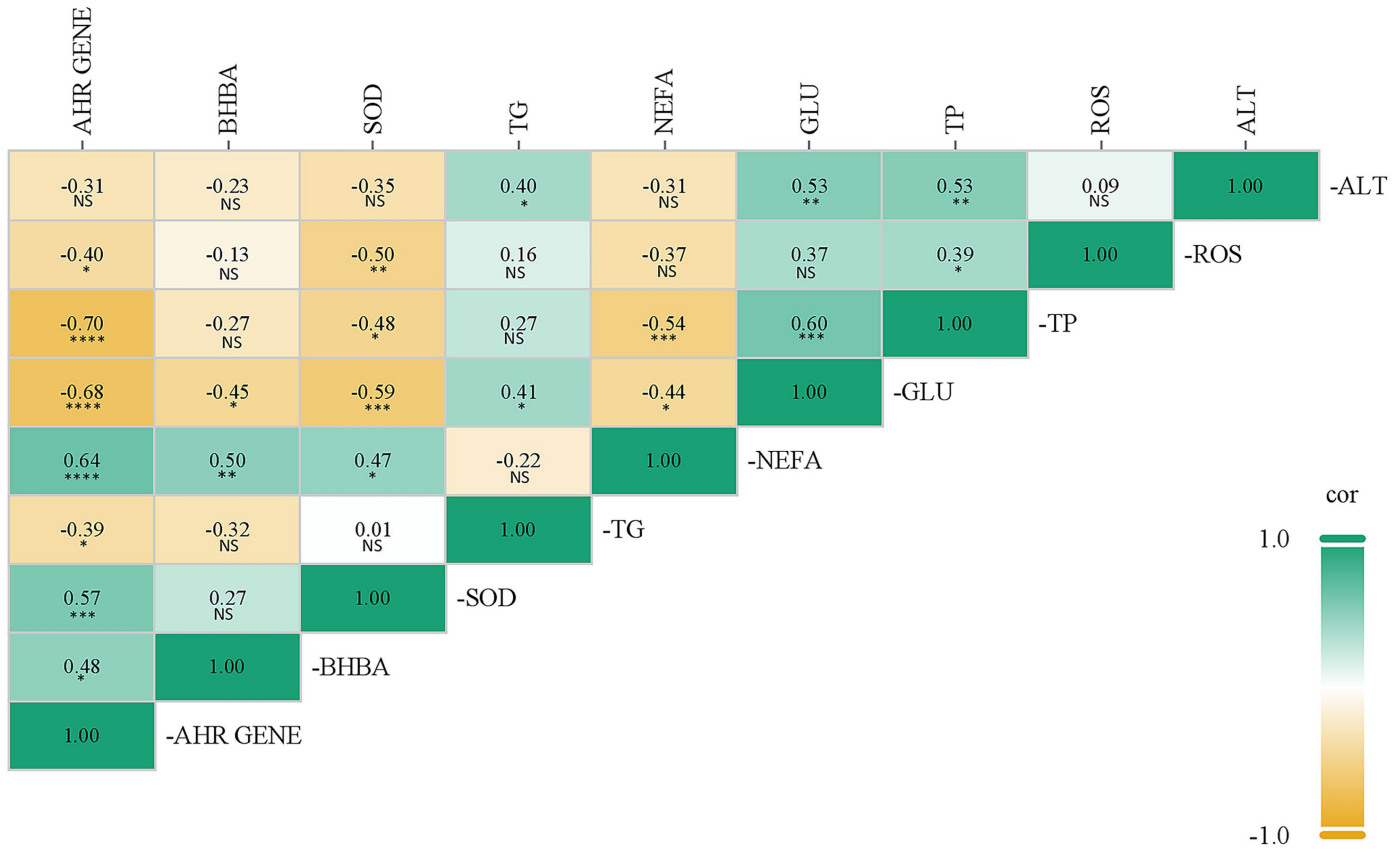
Correlation analysis of *AHR* gene with blood key biochemical indicators, antioxidant indicators. The corresponding values of the correlation index are indicated in the grid, and the color of the grid represents the level of the correlation index. The graph comes with a *p*-value determination function. The determination criteria are as follows: *p < 0.05, **p < 0.01; ***p < 0.005, ****p < 0.001 and NS indicates no significant difference (*p >* 0.05). All data are presented as “Mean ± SD.”

This study employed Spearman correlation analysis to conduct a correlation analysis between the *AHR* gene and blood biochemical indicators and antioxidant indicators, in order to further reveal the correlation between the expression of the *AHR* gene and phenotypic indicators in dairy cows. The results of the correlation analysis showed that there was a highly significant positive correlation between NEFA content and *AHR* gene expression (*p <* 0.001, *r* = 0.64), a highly significant negative correlation between GLU content and *AHR* gene expression (*p <* 0.001, *r* = −0.68), a highly significant negative correlation between TP content and *AHR* gene expression (*p <* 0.001, *r* = −0.70), directly link to the energy imbalance and disordered fat mobilization in ketosis. Furthermore, a highly significant positive correlation between SOD activity and *AHR* gene expression (*p <* 0.005, *r* = 0.57), a significant positive correlation between BHBA content and *AHR* gene expression (*p <* 0.05, *r* = 0.48), a significant negative correlation between TG content and *AHR* gene expression (*p <* 0.05, *r* = −0.39), and a negative correlation between ALT activity and *AHR* gene expression, with no significant difference (*p >* 0.05, *r* = −0.31) (Figure 7).

The expression of the *AHR* gene exhibited a positive correlation with NEFA and BHBA levels, and a negative correlation with GLU and TG levels. These correlations directly reflect the energy imbalance and disordered fat mobilization central to ketosis pathogenesis. Furthermore, its negative correlation with TP and positive correlation with SOD activity suggest the involvement of *AHR* in ketosis-related liver function impairment and the body’s adaptive antioxidant response. Collectively, these findings highlight the value of *AHR* as a molecular marker that integrates multiple pathological pathways of ketosis.

## 4 Discussion

### 4.1 Summary of key findings

This study aimed to investigate the pathogenesis of ketosis at the genetic level and to establish an early-warning method. While recent advances have demonstrated the feasibility of non-invasive monitoring approaches—such as the use of milk mid-infrared (MIR) spectroscopyto predict blood metabolites like NEFA and BHBA for herd-level metabolic screening—these tools primarily reflect phenotypic outcomes and may lack the resolution needed for individual-level diagnosis or mechanistic insight (15). In this context, our work focuses on uncovering the molecular underpinnings of ketosis. Blood biochemical indicators (NEFA, GLU, ALT, TG, TP) and antioxidant markers (ROS, SOD) were identified as potential early-warning indicators. Notably, transcriptome analysis revealed numerous differentially expressed genes, from which the *AHR* gene was selected as a candidate molecular marker for ketosis prediction. Subsequently, a qPCR-based detection method was successfully developed, demonstrating high specificity, sensitivity, and repeatability.

### 4.2 Screening of differential biochemical indicators of blood

Ketosis in Dairy cow is commonly associated with elevated NEFA levels, which is a typical manifestation during specific physiological stages, such as the dry and early lactation periods. When energy intake becomes insufficient to meet the cow’s requirements, the body mobilizes fat stores to compensate, leading to an increase in plasma NEFA levels. GLU and TG are two key factors in abnormal liver metabolism. Insufficient GLU uptake in ketotic cows’ triggers excessive mobilization of body fat, causing NEFA to be esterified into TG in the liver (16). This study showed that TG content in serum during the pre-perinatal period was significantly higher than in the postpartum period, a change closely linked to the physiological shifts occurring as cows enter the lactation period. When TC is in metabolic balance, NEB accelerates fat mobilization, increasing plasma TC levels. TC exists primarily in three forms: HDL-C, LDL-C, and VLDL. LDL-C transports cholesterol to extrahepatic tissues, but when excessive NEFA is esterified into LDL-C and deposited in the liver, the liver’s metabolic capacity declines, increasing ketone body production (17). In this study, compared to the PHC group, serum TC levels in the HC group significantly increased, TG levels significantly decreased, and LDL-C levels remained unchanged. This suggests that VLDL and HDL exchange substances in the blood, hydrolyzing TG and producing LDL-C, maintaining LDL-C balance. In the CK group, both TG and LDL-C levels decreased, indicating that increased ketone body content is associated with altered liver cholesterol metabolism. These findings align with the results of Xu et al. (18), who reported increased fat mobilization in ketotic cows 13 ± 5 days postpartum, resulting in decreased LDL-C levels. AST, ALT, and GGT are plasma indices closely linked to liver damage in ketotic cows. This study found no significant differences in GGT activity among groups, consistent with the findings of Mohsin et al. (19), suggesting that GGT alone may not serve as a sensitive indicator of ketosis. In ketotic cows, NEB aggravates fat mobilization and increases liver fat deposition, damaging functional liver cells and releasing ALT and AST into the bloodstream, thereby elevating enzyme activity (20). This study observed significantly increased AST levels in all postpartum cows, confirming that AST activity rises in ketotic cows (21). However, it is noteworthy that ALT activity is not exclusively liver-specific. Studies indicate that in ketotic cows, AST activity generally increases, while ALT activity remains unchanged or slightly elevated. In this study, ALT levels in ketotic cows were significantly lower than in healthy cows, potentially due to other influencing factors. Some studies suggest that subclinical ketotic cows may also exhibit lower serum ALT levels than healthy cows (22), highlighting the complexity of ALT activity in ketosis. Generally, GGT levels in ketotic cows are higher than in healthy cows. By analyzing AST, ALT, and GGT levels, this study identified liver injury in ketotic cows, corroborating findings by Du et al. (23), who observed significant increases in AST and GGT levels in ketotic cows.

NEB in dairy cows also reduces feed intake, limiting amino acid absorption and subsequently decreasing ALB synthesis. TP and ALB are critical liver function indicators. Under ketosis, fatty degeneration of the liver often occurs, leading to significant decreases in TP and ALB levels in the CK group compared to the PHC group. This decrease reflects impaired liver function.

Additionally, significant differences in LDH content were observed between the PCK and CK groups and between the PHC and HC groups. This may be attributed to increased energy demands and limited oxygen supply during labor, prompting cells to increase anaerobic glycolysis, thereby elevating lactic acid production.

In conclusion, monitoring ALT, TG, and TP levels in dairy cows can facilitate the timely detection and prevention of ketosis. The NEB state in dairy cows is typically associated with elevated NEFA levels, which are common during specific physiological stages such as the dry and early lactation periods (24). When energy intake fails to meet demands, the body mobilizes fat reserves, leading to increased plasma NEFA levels (25). Additionally, when acetyl-CoA produced by NEFA oxidation in the liver cannot be fully utilized by the TCA cycle, it is converted into ketone bodies, potentially triggering ketosis in severe NEB states (26).

GLU serves as a vital energy source for perinatal dairy cows, supporting fetal growth, lactation, and normal physiological functions. It is also a key indicator for assessing NEB in dairy cows (27). In this study, compared with the prepartum group, the CK group exhibited significantly increased BHBA levels, while these levels remained stable in the healthy group. The NEFA levels in the CK group were higher than those in the PCK and HC groups, and postpartum GLU levels significantly declined across all groups, with notable differences between the CK and HC groups. These findings highlight the diagnostic significance of BHBA, NEFA, and GLU in identifying postpartum ketosis. However, prenatal BHBA levels showed no significant difference, rendering it unsuitable as a predictive marker for ketosis before delivery, possibly due to varying physiological stages and nutritional states. Prenatal increases in NEFA and decreases in GLU, however, emerged as potential indicators for predicting ketosis before delivery.

### 4.3 Screening of differential antioxidant indicators in blood

The immuno-antioxidant capacity of perinatal dairy cows changes significantly and is closely linked to health and production performance. Oxidative stress, indicated by increased ROS levels, is a physiological state resulting in cell and tissue damage (28). In this study, ROS levels in the PHC group were significantly higher than those in the PCK and HC groups, suggesting that oxygen free radical levels may serve as an early warning indicator of ketosis. MDA, a product of lipid peroxidation and an important oxidative stress marker, was higher in prepartum groups than postpartum groups, indicating reduced oxidative stress levels postpartum (29, 30).

The antioxidant defense system in postpartum dairy cows showed significant improvement, encompassing both enzymatic and non-enzymatic mechanisms that scavenge ROS and reduce MDA levels. Enzymatic antioxidants such as SOD, CAT, and GSH-Px exhibited upward trends postpartum. SOD converts superoxide anions into hydrogen peroxide (31), CAT breaks down hydrogen peroxide into water and oxygen, and GSH-Px oxidizes glutathione to reduce hydrogen peroxide or lipid peroxides (32) Among these, SOD levels were significantly higher in the PCK group compared to the PHC group before delivery, supporting its potential as an early warning marker for ketosis.

INS sensitivity may be altered in perinatal dairy cows, weakening their response to INS and affecting energy balance, oxidative stress, and inflammatory responses (33). These changes are significant mechanisms underlying metabolic syndromes such as ketosis and obesity (34, 35). This study observed a significant decrease in INS levels in the CK group. The reduction in INS may result from:decreased GLU levels reducing stimulation of islet B cells, leading to reduced INS secretion. Increased protein demands during late pregnancy, diminishing INS synthesis. Fat mobilization to balance energy needs, which elevates NEFA and BHBA levels, inhibiting INS secretion (35).

### 4.4 Transcriptome analysis and identification of key genes

This study effectively identified DEGs across different groups, revealing potential involvement in key signaling pathways and regulatory mechanisms underlying ketosis in dairy cows. A total of 907 upregulated genes and 246 downregulated genes were identified in the PCK vs. PHC comparison. Among these, the *AHR*, *RASGRP3*, and *KMT2A* genes exhibited the most significant expression differences, suggesting their pivotal roles in ketosis.

The *AHR* gene is associated with various biological processes, including cell metabolism, immune response, and cell proliferation (36). The *RASGRP3* gene is linked to signal transduction pathways, while the *KMT2A* gene is involved in transcriptional regulation (37, 38). The significant changes observed in these genes may reflect the molecular basis of dairy cows’ susceptibility to ketosis in the pre-delivery stage, making them promising candidate genes for predicting ketosis.

In the PCK vs. PHC comparison, DEGs were significantly enriched in 44 KEGG pathways, primarily related to metabolism and energy balance, immune system function, and inflammatory responses. These findings suggest that ketosis may arise from disruptions in these biological processes. Notably, ketosis in dairy cows is closely associated with the Cushing’s syndrome metabolic pathway. Cushing’s syndrome, characterized by prolonged exposure to excessive glucocorticoids (GCs), disrupts normal metabolic processes, causing insulin resistance, increased fat breakdown in adipose tissue, and enhanced gluconeogenesis in the liver (39, 40). GCs can also increase visceral adipose tissue, elevate circulating NEFA levels, and exacerbate insulin resistance and metabolic disorders (41, 42).

This study found that highly expressed DEGs in the PCK group, such as *KMT2A, ITPR2*, *AHR*, and *ITPR1*, were involved in regulating the Cushing’s syndrome metabolic pathway. Understanding the roles of these genes in this pathway may provide insights into the molecular basis of ketosis and inform the development of new early prevention strategies.

In the PCK vs. CK comparison, 1,165 DEGs were identified. GO enrichment analysis highlighted significant enrichment in biological process (BP) terms, such as immune response and calcium ion signal transduction, reflecting immune regulation changes in the postpartum stage. Cellular component (CC) enrichment emphasized roles in the cell membrane and cellular structures, while molecular function (MF) enrichment pointed to key mechanisms like chemokine binding and MAP kinase activity. DEGs were enriched in 37 KEGG pathways, involving immune response, cell signaling, metabolism, endocrinology, and disease-related processes, emphasizing the influence of immune, metabolic, and endocrine systems on ketosis.

In the CK vs. HC comparison, 345 DEGs were identified, reflecting adaptive changes in the mRNA transcriptome in response to postpartum ketosis. GO enrichment analysis linked these DEGs to cell proliferation, migration, and division, involving centrosomes, actin cytoskeletons, and receptor binding. KEGG analysis revealed enrichment in five pathways, including metabolism, endocrinology, immune response, and disease pathways.

Although the etiologies of dairy cow ketosis and type I diabetes differ, both conditions share disruptions in insulin function and glucose metabolism (43). Insulin resistance in dairy cows with ketosis may result from the inhibitory effects of high ketone body and NEFA concentrations on insulin signaling (44), paralleling insulin resistance and deficiency in type I diabetes (45).

In the PHC vs. HC comparison, 977 DEGs were identified. GO enrichment analysis highlighted significant enrichment in BP terms, including calcium ion-mediated signaling, heme biosynthesis, and inflammatory response. KEGG analysis identified 23 enriched pathways related to signal transduction, metabolic pathways, substance synthesis, and cellular processes. These results revealed critical changes in the biological processes of healthy and diseased states, providing a foundation for understanding physiological regulation and metabolic maintenance in dairy cows.

This analysis offers new perspectives and directions for future research, contributing to the improvement of dairy cow welfare and production efficiency.

### 4.5 The potential of the *AHR* gene as a molecular marker

*AHR* is a ligand-activated transcription factor, and its activation in cells is closely associated with the regulation of various biological effects (46, 47). In this study, the *AHR* gene demonstrated significant changes during the DEG screening between the PCK and PHC groups. Transcriptomic analysis revealed a log2FoldChange value of 2.05 and a *p*-value of 9.17 × 10^−5^, indicating a significant difference in *AHR* gene expression levels between these groups. Furthermore, qPCR validation showed a 4.02-fold difference in the relative expression of the *AHR* gene, supporting its potential role in the development of ketosis.

Among the DEGs identified, including *RASGRP3* and *KMT2A*, *AHR* was prioritized as the prime candidate molecular marker for several reasons. First, *AHR* exhibited not only statistically significant overexpression in pre-ketotic (PCK) cows but also the most consistent and robust correlation with the onset of ketosis, as evidenced by its central role in the key co-expression module (Blue module) identified by WGCNA. Second, functional analyses provided a compelling biological rationale: KEGG pathway analysis indicated that *AHR* is involved in the regulation of the Cushing’s syndrome-related metabolic pathway, which shares core pathophysiological features with ketosis, such as energy imbalance and disordered fat metabolism. Moreover, *AHR* showed a strong association with the Th17 cell differentiation pathway, suggesting its involvement in the immunometabolic dysregulation characteristic of ketosis. Third, correlation analysis demonstrated that *AHR* expression levels were integrally linked to multiple core pathological processes of ketosis, showing significant correlations with key biochemical indicators (NEFA, GLU, TP) and antioxidant markers (SOD). While other DEGs like *RASGRP3* and *KMT2A* were also significant, their functional connections to ketosis-specific pathways were less comprehensive compared to *AHR*, which appears to be a nexus gene integrating metabolic, oxidative, and immune disturbances. Although a combination of multiple DEGs might theoretically offer enhanced predictive power, the selection of a single, highly integrative marker like *AHR* was justified for developing a simple, rapid, and cost-effective field-applicable detection method. The high prediction accuracy achieved using *AHR* alone (91.11% in healthy cows) supports its practical utility. Future studies could explore multi-gene panels to further improve sensitivity and specificity.

### 4.6 Establishment and validation of transcription detection methods

In the dairy farming industry, the accurate and rapid diagnosis of ketosis is critical for maintaining animal health and improving production efficiency. Current diagnostic methods rely primarily on measuring ketone body levels in blood and milk, offering fast and accurate results suitable for on-site screening and routine monitoring. However, with the shift toward intensive dairy farming, there is an increasing demand for higher accuracy in early detection technologies for ketosis.

In this study, the *AHR* gene was identified as a candidate for early warning, and a qPCR-based detection method was developed using the dual standard curve method to measure *AHR* gene expression levels. The results showed that this detection method is simple, highly specific, and quantitatively accurate, with significant potential for the early detection of ketosis and developing prevention strategies for ketosis.

Testing 69 samples revealed that the positive predictive concordance rate for ketosis occurrence 2 weeks before delivery was 70.83%. The relative expression range of the *AHR* gene in these cases was 1.9571–15.5321, suggesting that significant pre-delivery changes in *AHR* expression levels are associated with an increased risk of ketosis. In negative control samples, the concordance rate for predicting healthy cows was as high as 91.11%, with *AHR* expression levels ranging from 0.4341 to 1.7924. This stability in healthy cows underscores the *AHR* gene’s utility as a predictive marker for ketosis.

### 4.7 Limitations and prospects

Future studies with larger sample sizes are needed to accurately define the critical threshold for *AHR* gene expression, which will facilitate the development of a more precise diagnostic tool for dairy cow ketosis. Furthermore, the specific molecular mechanisms through which *AHR* influences ketosis, particularly how it mediates energy imbalance via the regulation of immune-metabolic pathways such as Th17 cell differentiation, warrant further in-depth investigation.

## 5 Conclusion

The experimental results demonstrated that changes in NEFA, GLU, ALT, TG, TP, ROS, and SOD levels before delivery could serve as diagnostic indicators for early warning of ketosis. Additionally, transcriptomic analysis identified the *AHR* gene as a potential candidate for predicting dairy cow ketosis. A qPCR-based *AHR* gene detection method for early warning of ketosis was successfully established, with highly specific primers, a minimum detection limit of 10 copies/μL, and 100 times greater sensitivity compared to conventional PCR technology. This method exhibited good repeatability, a positive predictive concordance rate of 70.83%, and a prediction accuracy of 91.11% for healthy cows. The detection method has significant potential for application in predicting and managing ketosis in dairy cows.

## Data Availability

The transcriptome data of dairy cow blood has been deposited in the NCBI database with the accession number PRJNA1416680.
